# Rough Surface Characterization Parameter Set and Redundant Parameter Set for Surface Modeling and Performance Research

**DOI:** 10.3390/ma15175971

**Published:** 2022-08-29

**Authors:** Duo Yang, Jinyuan Tang, Fujia Xia, Wei Zhou

**Affiliations:** 1 State Key Laboratory of High Performance Complex Manufacturing, Central South University, Changsha 410017, China; 2 College of Mechanical and Electrical Engineering, Central South University, Changsha 410017, China; 3 Hunan Provincial Key Laboratory of High Efficiency and Precision Machining of Difficult-to-Cut Material, Hunan University of Science and Technology, Xiangtan 411199, China

**Keywords:** roughness surface, morphological characterization, characterization parameter set, redundant parameter set, ISO25178

## Abstract

Among the 26 roughness parameters described in ISO 25178 standard, the parameters used to characterize surface performance in characterization parameter set (**CPS**) lack scientificity and unity, resulting in application confusion. The current **CPS** comes from empirical selection or small sample experiments, thus featuring low generality. A new method for constructing **CPS** in rough surfaces is proposed to solve the above issues. Based on a data mining method, statistical theory, and roughness parameters definitions, the 26 roughness parameters are divided into **CPS** and redundant parameter sets (**RPS**) with the help of reconstructed surfaces and machining experiments, and the mapping relationships between **CPS** and **RPS** are established. The research shows that **RPS** accounts for 50%, and **CPS**, of great significance for surface performance, and has the ability to fully cover surface topography information. The birth of **CPS** provides an accurate parameter set for the subsequent study of different surface performance, and it provides more effective parameters for evaluating the workpiece surface performance from the same batch.

## 1. Introduction

With the rapid development of the modern precision manufacturing industry, products have shifted from macroscopic shape control to microscopic shape collaborative design and manufacturing. Surface roughness, as a part of the micro-structural information, can significantly affect surface sealing, wear, fatigue, etc. [1,2,3]. In this sense, it is of great significance to carry out surface roughness characterization research [4]. Due to the complex information covered by surface roughness, researchers have tried to formulate some roughness parameters to describe different surface morphology characteristics. Therefore, surface roughness characterization is transformed into a correlation study among roughness parameters.

In 1929, G. Schmalz in Germany was the first to propose the surface roughness measurement baseline and evaluation coefficients, which provided an effective index to open the era of quantitative evaluation in surface roughness [5]. Subsequently, by constructing the curve between the surface peak-valley depth and the bearing length rate, Abbott formulated some roughness parameters to display the surface roughness information from different angles [6]. After that, other countries began to define their national surface roughness standards on the basis of their industrial application scenarios and actual production needs. The symbols and definitions of the parameters are of great difference, thus resulting in bad situations such as small application range, low reliability and versatility, the phenomenon of “parameter big bang” [7]. The number of parameters reached more than one hundred at one time [8] in standards.

In order to unify roughness parameters standards, the technical committee of the International Organization for Standardization (ISO) carried out a revision of the surface topography standard specification in 1996, and divided roughness parameters into seven categories based on the definitions [9]. However, the definitions of two-dimensional (2D) roughness parameters were based on the root mean square of the profile section height and could only cover the surface characteristic information in the X and Y directions. It inevitably led to the loss of topographic information and could not achieve a good characterization of three-dimensional (3D) roughness surface.

With the development of surface topography measurement and computer digital technology, the lossy stylus measurement in surface roughness was gradually replaced by the non-destructive optical measurement. This great improvement provided reliable technical support for the birth of 3D surface roughness parameters based on topography mid-surface. The University of Birmingham took the lead in defining 3D roughness parameters, later called “Birmingham 14 parameters” [10], to lay the foundation for subsequent surface roughness standards. In 2010, the ISO extended the “Birmingham 14 parameters” to ISO 25178 3D roughness parameters standards with the help of two-dimensional standards and topography measurement technology, and the categories were upgraded to six: height parameters, spatial parameters, hybrid parameters, functional parameters, volume parameters, and feature parameters [11,12].

However, the increasement and extension from 2D to 3D led to complex internal correlations and a low degree of matching between their definitions and categories. Some parameters even own repeated characterization information. This means that some of 3D roughness parameters in ISO 25178 are not developed through rigorous research and do not actually contain valid information to characterize surface performance. As roughness parameters are widely used in the study of surface performance and once there is some deviation in the selection of roughness parameters or the roughness parameters themselves do not have valid information, the results will be unreliable. It is key to establish a reasonable characterization method to find the roughness parameters suitable for the subsequent research of various surface performance.

In recent years, researchers have carried out internal correlation analysis in 3D roughness parameters and explored the correlation between 3D roughness parameters and surface performance. For example, Franco studied the correlation of S-series and V-series parameters in ISO 25178 standard, and believed that there was information redundancy between ***Spk*** and ***Vmp*** [13]. Pawlus carried out the study on the correlation of two-dimensional roughness height parameters, and elucidated that there was a strong positive correlation between ***Ra*** and ***Rq*** [14]. Qi et al. used the Spearman rank correlation coefficient to judge the internal correlation of the six categories in 3D roughness parameters, and established the parameter rank tree to distinguish the correlation strength [15]. However, the above work either involves an incomplete number of parameters, or the correlation analysis method just fits in dividing the roughness parameters according to the correlation level and cannot accurately distinguish the correlation strength in the same level.

Therefore, some researchers tried to select reasonable 3D **CPS** based on surface performance and parameter application frequency. M. Sedlaček et al. studied the correlation of ***Sq***, ***Ssk*** and ***Sku*** in the height parameters with the surface friction coefficient and clarified the influence of these three roughness parameters on the friction performance [16,17,18]. B. He et al. found the relationships between the critical load and 3D roughness parameters ***Spc***, ***Sq***, ***Vvc***, ***Sdq*** in the micro-connected structure, and gave application range of ***Spc***, ***Sq***, ***Vvc***, ***Sdq*** [19]. Zeng et al. combined the surface height probability density function, autocorrelation function and height parameters to evaluate the correlation of 2D and 3D roughness parameters to surface bearing capacity, friction and lubrication performance [20]. Todhunter analyzed the industry application of different roughness parameters by collecting the utilization frequency of the parameters from a total of 179 industrial users in 34 countries [21]. However, the method of selecting target roughness parameters only by engineering experience or application frequency lacks effective theoretical support. Besides, due to the large number of application scenarios and various surface properties (friction, wear, fatigue, lubrication, sealing, etc.), the performance screening method still results in “parameter big bang” to characterize surfaces. Therefore, it is urgent to design an effective characterization method to unify the selection range of target roughness parameters under different properties and ensure that there is no information redundancy in selection to avoid the deviation of research objectives.

It is generally acknowledged that each surface performance parameter is closely influenced by the surface geometry. Therefore, taking the surface geometry characterization research as the start, the roughness parameter characterization set will be established to characterize different performance parameters with full potential. Although there exist different definitions and expressions in ISO 25178, the data trend analysis shows that some roughness parameters fluctuate with others, and thus the information of these parameters is actually expressed by other parameters. If roughness parameters expressed by others are regarded as the “redundant parameter sets (**RPS**)” and the rest are classified as the “characterization parameter sets (**CPS**)”, the comprehensive characteristics description of the surface topography by **CPS** will be realized with the removal of redundant information.

Combined with the data feature, Pearson correlation analysis is used to roughly delineate **CPS** and **RPS** selection. With the principle of statistical non-strong and non-weak introduced [22], the core roughness parameters (**CRP**) used to characterize different redundant parameters are selected from **CPS**. Based on the current situation that the polynomial regression model is prone to lead to redundant items, a new method of item number reduction is proposed to construct the optimal explicit expressions between **CRP** and **RPS**, which realizes the information coverage from **CRP** to **RPS**. The optimal explicit expression can automatically find roughness parameters with strong characterization and furtherly determine **RPS** and **CPS**. Finally, the research verifies the reliability of the optimal explicit expression by the reconstruction and the measured surfaces. The designed characterization method can provide the guidance and basis for selecting reasonable target **CPS** for industrial applications.

## 2. Basic Concepts and Research Methods

The overall method technical route is in Appendix A. The followings are the detailed introductions.

(1) Due to the large number and complex correlation of roughness parameters, and the instability under small samples, the paper uses the stochastic process theory and surface reconstruction technology to set the value interval of seven reconstruction coefficients, and randomly combines them in their respective intervals. Finally, 1000 sets of reconstructed surfaces are generated for the research data and it ensures the reliability (Section 2.3 for details);
(2) 1000 groups of surface roughness parameters are obtained by ISO25178 definitions, and six types of roughness parameters are initially divided into **CPS** and **RPS** by combining Pearson correlation analysis and non-strong and non-weak statistical principles. Considering that a single redundant parameter should not be characterized by all the parameters in **CPS**, **CRS** characterizing different redundant parameters are selected from **CPS** and the correctness of parameter sets is analyzed based on the parameters definitions (Section 2.2 and Section 3.1 for details);
(3) The process in step 2 still cannot clear the quantitative characterization relationships between **CPS** and **RPS**. By means of polynomial regression model with strongly nonlinear characterization ability, a standard deviation of automatic pruning method is born. The method can automatically find roughness parameters of the surface with strong characterization ability and eliminate irrelevant factors. It realizes the explicit formula expression from **CPS** to **RPS** and uses the experiment to prove that **CPS** can cover the rough surface information (Section 2.4, Section 3.2 and Section 3.3 for details);
(4) To illustrate the engineering significance of **CPS** on surface performance, the paper describes the application of **CPS** in surface performance research by means of roughness parameter definition, neural network, sensitivity analysis, optimization algorithm, finite element calculation, etc. The reliability of these applications is discussed by the existing research, which provides the direction for the **CPS** application, and clarifies the practical significance (Section 3.4 for details).

### 2.1. 3D Roughness Parameter

Due to the complexity of 3D roughness surface information, ISO 25178 has defined a total of 26 main roughness parameters in six categories to characterize the surface roughness and describe the different topography features. The detailed definitions and descriptions can be found in literature [6,10]. The paper only briefly introduces their relevant symbols and definitions in Table 1.

### 2.2. Definition of Parameter Set

As the definitions and expressions of 3D roughness parameters vary greatly and some of them are defined based on experience, it is often difficult to analyze their internal correlation from definitions or formulas, which becomes the key point that has puzzled researchers for many years [22]. Differences always lie in some parameter formulas, but there exists an obvious data fluctuation trend between them. For example, there are great differences between the formulas of ***Sa*** and ***Sq***, but the research shows that they follow a linear trend with nearly 0.8 times between the two [23,24]. It reflects the fact that the information of some redundant parameters is actually represented by other parameters among the ISO 25178 standard, thus causing the application confusion in industrial production.

Therefore, for the sake of clarifying the quantitative correlation, the research tries to use data mining method and statistical theory to illustrate the correlation of different roughness parameters and find the roughness parameter set that truly characterizes the surface topography based on the idea that some roughness parameters follow the fluctuations of others. In order to facilitate the development of the work, the concepts of “redundant parameter sets (**RPS**)”, “characterization parameter sets (**CPS**)”, and “core roughness parameters (**CRP**)” are proposed and introduced in the following.

(1) Redundant parameter sets

The redundant parameter set is defined as the parameter set characterized by other roughness parameters. Specifically, it includes the parameters that can be determined by explicit expressions from others, so as to complete the information coverage and remove redundant information;

(2) Characterization parameter sets

**Figure 1:**
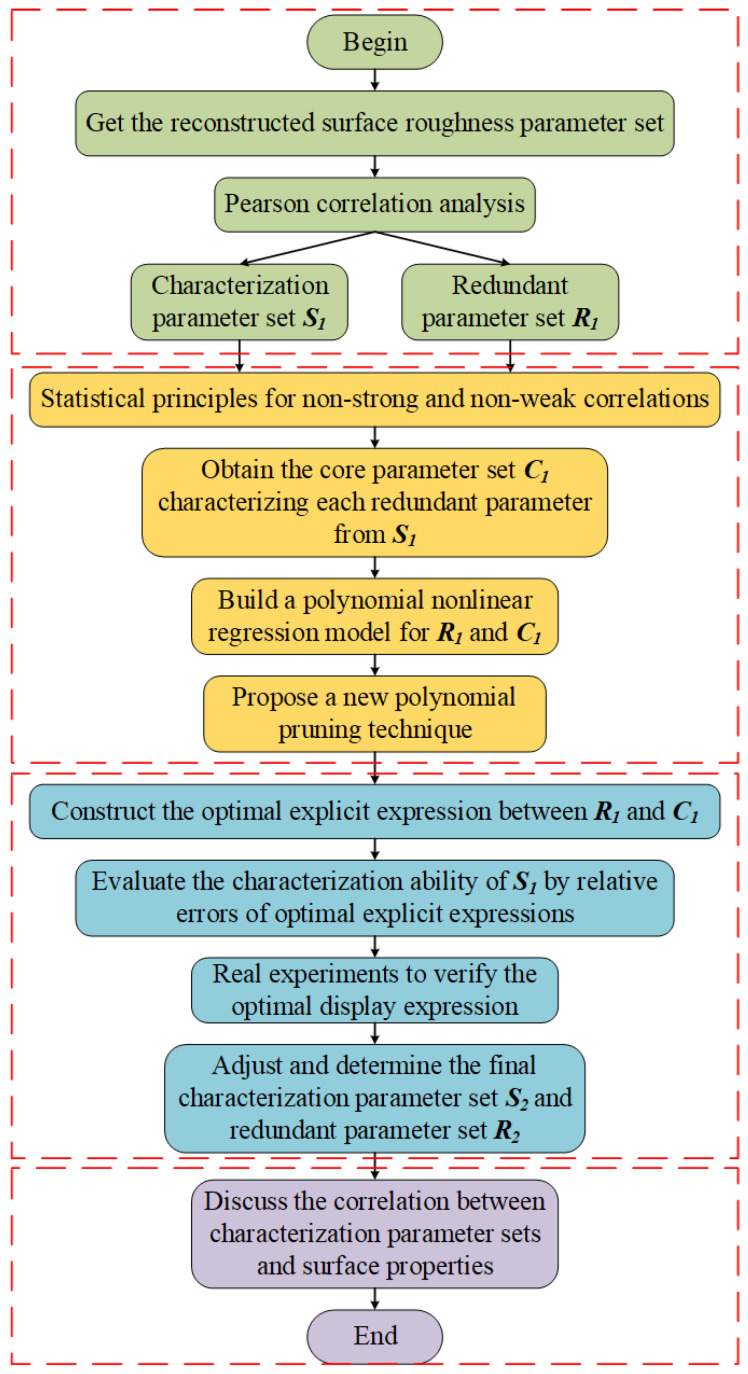
Construction of **CPS**.

The characterization parameter set is the parameter set used to predict **RPS**, and **CPS** is regarded as the set that truly characterizes the surface topography without redundant information, and is the research object with high correlation in different surface performance studies. Figure 1 shows the brief construction of **CPS**.

a. Based on the unclear correlation dilemma, Pearson correlation analysis is used to roughly distinguish the correlation between different roughness parameters. The all parameters are divided into **CPS** and **RPS**;
b. Owing to the large number of characteristic parameters in the initial division, it is not conducive to constructing the optimal explicit expression between **CPS** and **RPS**. Therefore, the statistics principle of non-strong and non-weak is introduced to screen **CRP** from **CPS** for the sake of characterizing each redundant parameter and then the parameter in **CRP** is regarded as the independent variable in the optimal explicit expression;
c. A new polynomial pruning method is proposed to establish the optimal explicit expressions between **CRP** and **RPS**. The relative error of the expression is used to evaluate the information reflection ability from **CRP** to **RPS**, and finally the reliability of **RPS** and **CPS** is verified and adjusted based on real experiments;
d. After the reliability of **CPS** is verified based on theory and experiments, the correlation between the **CPS** and surface performance parameters is extended and analyzed to point out the practical significance of **CPS** in engineering research.

(3) Core roughness parameters

The core roughness parameters are parameters from **CPS**, furtherly used as the independent variables of the optimal explicit expression.

### 2.3. Surface Reconstruction

As a result of the complex internal correlation among surface roughness parameters, it is difficult to obtain accurate correlation if the number of research samples is small in characterization. A reliable parameter characterization method needs to be built on the analysis with a large number of surface samples to ensure its generality and stability. The large number of real surfaces experiments, especially at the early stage of the research, will result in a high cost of trial and error for researchers. And if the research is just based on experiments, it will easily lead to a sudden increase in time and money costs.

The utilization of numerical method to generate reconstructed surfaces with different roughness characteristics can avoid the above problems and provide a large number of surface samples in a short time. The grinding surface is the common representative one among non-Gaussian surfaces, so the paper takes the surface with grinding characteristics as the object. Its surface reconstruction is generally based on the surface height probability density function and the autocorrelation function. The following is the brief introduction to the reconstruction surface method.

According to the Johnson transformation method [25], the height probability density function can be obtained by three characteristic coefficients, while the autocorrelation function is controlled by four characteristic coefficients. These coefficients are defined as follows:

(1) Characteristic coefficient of height probability density function

The three characteristic coefficients *k*_1_, *k*_2_ and *k*_3_ [26] of the height probability density function are the second, third and fourth order center distances of the reconstructed surface, respectively:

(1) $$k_{1}=\frac{1}{N}{\sum \left(Z-\bar{Z}\right)}^{2}$$

(2) $$k_{2}=\frac{1}{N}{\sum \left(Z-\bar{Z}\right)}^{3}$$

(3) $$k_{3}=\frac{1}{N}{\sum \left(Z-\bar{Z}\right)}^{4}$$

Here, *Z* is the surface height matrix; $\bar{Z}$ is the mean value of *Z*; *N* is the number of elements in the matrix.

(2) Characteristic coefficient of autocorrelation function

The four coefficients *a*_1_, *a*_2_, *a*_3_ and *a*_4_ of the autocorrelation function are shown in Formula (4) [27]:

(4) $$C=\left[a_{1}e^{-a_{2}\tau_{x}}+\left(1-a_{1}\right)\cos\left(a_{3}\tau_{x}\right)\right]e^{-a_{4}\tau_{y}}\;$$

Here, *C* is the surface autocorrelation function.

After the coefficients of the surface height probability density function and the autocorrelation function have been obtained, the random matrix ***R*** can be generated by combining the random process theory [28] and the fast Fourier transform method.

(5) $$R=\mathrm{ifft}2\left(e^{i2\pi \phi }\right)$$

Here, ifft2() represents the inverse Fourier transform of the matrix; *ϕ* is the characteristic function of the fast Fourier transform.

After that, *C* will be expanded into the autocorrelation function matrix *C*′ through the symmetry assumption with the random matrix ***R*** [29]. By further applying the transformation to the random matrix R and the autocorrelation function matrix *C*′ with the following formula, the height matrix *Z_f_* of the grinding surface can be obtained.

(6) $$Z_{f}=\frac{\mathrm{fft}2\left(C^{\prime }\right){{}^{\circ}}^{1/2}\mathrm{fft}2\left(R\right)}{\left|\mathrm{fft}2\left(R\right)\right|}$$

Here, fft2() represents the Fourier transform of the matrix; ° means that each element of the matrix is operated separately.

**Figure 2:**
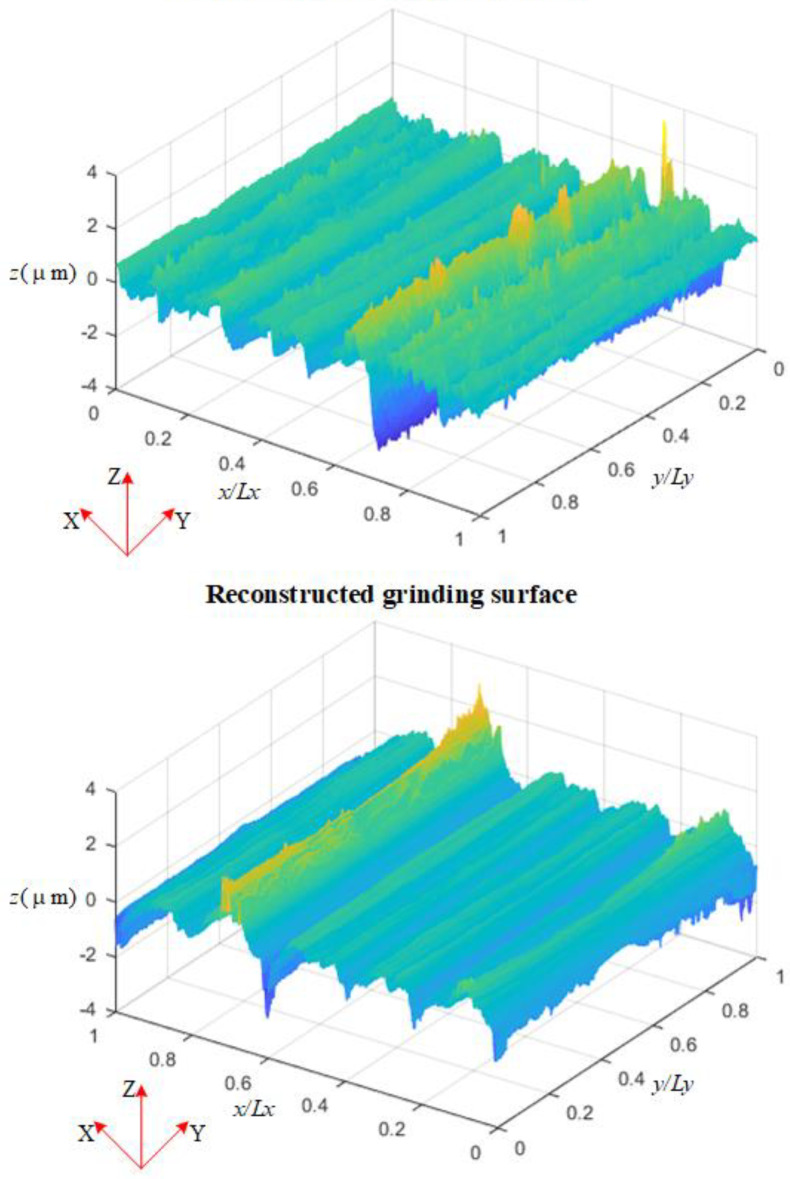
Measured and reconstructed grinding surface topography.

Figure 2 displays the schematic diagram of the real measurement grinding surface and reconstructed grinding surface. The reconstructed surface has obvious grinding texture characteristics.

### 2.4. Principle of Optimal Explicit Expression

Since the correlation of roughness parameters is difficult to explore theoretically from the perspective of definition, the correlation model between **RPS** and **CPS** often stands on BP neural network or nonlinear regression model. However, BP neural network is not fit to provide a simple and explicit expression with less ability to realize the practical application in production. Therefore, the paper establishes the optimal explicit expressions between **RPS** and **CPS** by means of polynomial nonlinear regression model. **RPS** and **CPS** will be roughly distinguished by Pearson correlation analysis at first.

(1) Pearson correlation analysis

As roughness parameters belong to continuous data, the correlation among them should be studied through Pearson correlation analysis [30], and the calculation is as follows:

(7) $$r_{ij}=\frac{s_{ij}}{\sqrt{s_{ii}s_{jj}}}$$

*r_ij_* is the correlation coefficient between variable ***i*** and variable ***j***;
*s_ij_* is the covariance between variable ***i*** and variable ***j***;
*s_ii_* and *s_jj_* are the variances of variable ***i*** and variable ***j***, respectively.

(2) Polynomial nonlinear regression model

The polynomial nonlinear regression model is the data method with strong nonlinear fitting ability. It is applicable to the situation where the correlation between variables is not clear, so as to explore the influence of variables and realize the explicit expression of independent variables to dependent variables [31,32]. Here explains how it works:

Regard the dependent variable as a complete polynomial combination of ***n*** independent variables:

(8) $$\begin{array}{ll} f\left(x\right)= & a_{0}+a_{1}x_{1}+\cdots +a_{n}x_{n}+a_{n+1}x_{1}^{2}\\ & +a_{n+2}x_{1}x_{2}+\cdots +a_{N-1}x_{k}^{m}=\sum_{i=0}^{N-1}a_{i}u_{i}(x) \end{array}$$

Here,

$u_{i}\left(x\right)\;$is the complete polynomial under the *m* power of independent variables $x=\left(x_{1},x_{2},\ldots ,x_{n}\right)$;

$a_{i}\;$is the undetermined coefficient corresponding to the complete polynomial;

The total number of model terms is N=(n+m)!/(n!m!);

However, when the polynomial nonlinear regression model is directly used for the research, based on the total number of items in the model, the increase of the independent variables will lead to an explosive growth of the items, and it is easy to produce many redundant terms. These redundant terms are meaningless to improve the accuracy of the model, so the number of terms should be reasonably pruned [32,33] to establish the optimal explicit expression.

(3) Item number pruning method

**Figure 3:**
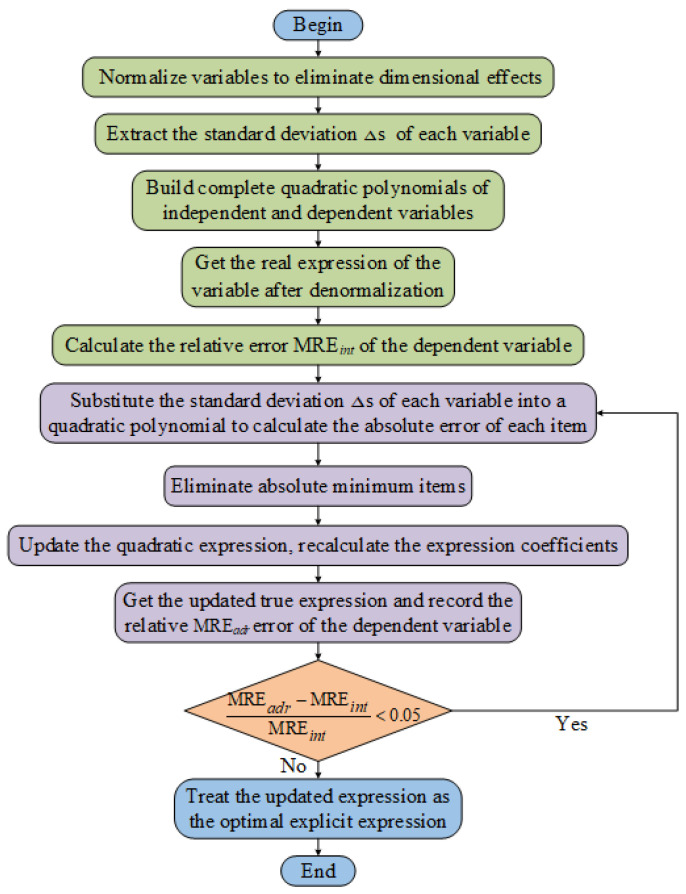
Optimal explicit expression screening process.

Aiming at the situation that the polynomial nonlinear regression model is prone to generating redundant items, the research designs a new method of reducing items and establishes the optimal explicit expressions between **RPS** and **CPS**. Its specific calculation is shown in Figure 3.

I. In order to remove the errors caused by different variable dimensions, the variables are normalized, and the standard deviation Δs of each variable after normalization will be calculated;
II. Establish the complete quadratic polynomial expression between the independent variables and the dependent variables, obtain the true expression after denormalization of the variables, and then calculate the relative error of the dependent variable MRE*_int_*;
III. Substitute the standard deviation Δs of the independent variable into the quadratic polynomial expression, calculate the absolute value of each item, and then remove the item with the smallest absolute value. Take the remaining terms as the updated quadratic expression, and solve for the updated expression coefficients;
IV. Calculate the updated denormalized quadratic expression and record the relative error MRE*_adr_* of the dependent variable at this time. If
  $$\frac{{\mathrm{MRE}}_{adr}-{\mathrm{MRE}}_{int}}{{\mathrm{MRE}}_{int}}<0.05,$$
  go back to step **III** to solve again and update the expression. Otherwise, take the final expression as the optimal explicit expression.

## 3. Results and Analysis

Based on Li [27] and Yang [22] et al.’s research on the correlation judgement and surface reconstruction, the paper firstly sets the coefficients of the height probability density function and the autocorrelation function, shown in Table 2. After a random selection of each coefficient, 1000 groups of reconstructed grinding surfaces are generated for subsequent research.

### 3.1. Classification for CPS and RPS

As mentioned above, the correlation among 26 roughness parameters is complex with hugely different definitions and formulas, thus causing the troubles. Therefore, the paper tries to preliminarily judge the correlation between the roughness parameters from data analysis and determines the correctness and error of the results with the help of parameters definitions. Since the data type belongs to continuous data, Pearson correlation analysis is used to distinguish the correlation between the roughness parameters.

When the Pearson correlation coefficient of the bivariate is large, there is a strong correlation between the two parameters. Variable **A** will closely follow the change of the variable **B**. At this time, it can be considered that variable **A** is controlled by variable **B**. When the correlation coefficient is extremely small, variable **A** either has a curve trend with variable **B**, or there is no actual correlation between the two. At the moment, it is generally necessary to use the trend distribution diagram and definitions to assist in judgement.

The criterion for determining the correlation strength is shown in Table 3 [14,15,30].

As is known, ***Sa*** is widely used to characterize the surface quality in the application of surface characterization and ISO 25178. The research intends to use ***Sa*** as the first benchmark in **CPS**. The specific method is as follows:

(1) Take ***Sa*** as the benchmark (the first selected to **CPS**), and evaluate the correlation between the remaining parameters and ***Sa***. The parameters whose correlation coefficient with ***Sa*** is greater than 0.9 are put into **RPS**, and the parameter with the smallest coefficient is selected as the next one into **CPS**. The remaining are classified as the undetermined set;
(2) Treat the second selected parameter as the next benchmark, calculate the correlation coefficient between the second and each parameter in the undetermined set. Parameters with the coefficient greater than 0.9 are put into **RPS**, and the one with the smallest coefficient is selected as the third one into **CPS**. The remaining are still used as the updated undetermined set;
(3) With similar operation as step 2, **CPS** and **RPS**, two different rough parameter sets, are finally obtained until the item number in the undetermined set is 0.

**Figure 4:**
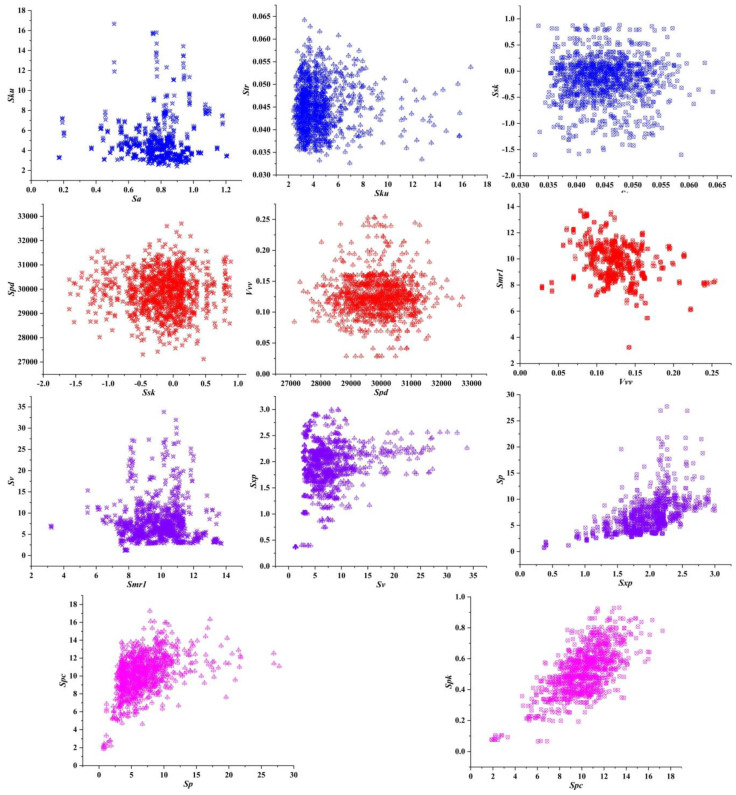
**CPS** scatter trend.

**Figure 5:**
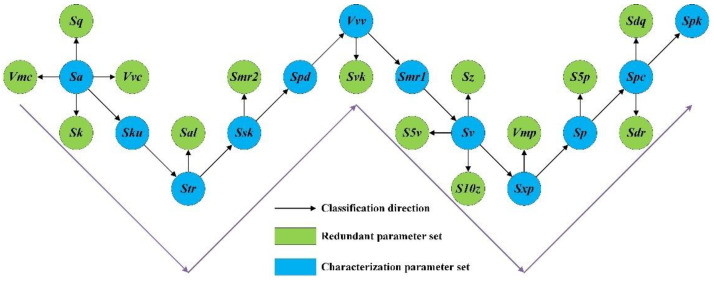
CPS and RPS.

The trend of each parameter in **CPS** during the screening process is shown in Figure 4. There is no obvious curve distribution of each parameter. Although ***Spc*** and ***Spk*** seem to present certain trend at the end of the screening, a careful observation reveals that in the same ***Spc***, the fluctuation of ***Spk*** accounts for 60% in the overall range, making it impossible to meet the standard that the correlation coefficient is greater than 0.9. Therefore, ***Spk*** enters into **CPS**. The specific screening process can be seen in Figure 5. **CPS** consists of ***Sa***, ***Ssk***, ***Sku***, ***Sp***, ***Sv***, ***Str***, ***Spk***, ***Smr1***, ***Sxp***, ***Vvv***, ***Spd*** and ***Spc***. The following is an introduction to the parameters in **CPS** to help determine the rationality of selection.

The above analysis shows that the method of distinguishing **CPS** and **RPS** with strong correlation not only illustrates the classification rationality from the perspective of data, but also proves the reliability of the discriminating method in combination with the physical meaning and definition of roughness parameters.

***Sa*** is the most widely used parameter, characterizing the average height difference in the surface. ***Sq*** which is highly related to ***Sa*** is classified into **RPS**. It is consistent with the work of Pawlus et al. [14]. While ***Ssk*** and ***Sku*** stand for surface skewness and kurtosis respectively, they are used to evaluate the symmetry and steepness of the surface height distribution. ***Ssk*** and ***Sku*** belong to the key factors of surface reconstruction theory [26] and are indispensable for surface characterization [34]. ***Sp*** and ***Sv*** represent the surface extremum features. Since Sp+Sv=Sz, the information of ***Sz*** is actually expressed by ***Sp*** and ***Sv***. Therefore, ***Sz*** is kicked into **RPS**. ***Str***, reflecting surface anisotropy and defined by the surface autocorrelation function, is usually used to characterize the surface texture direction and spatial information. According to the definitions, ***Sal*** and ***Str*** can iterate over each other, so information intersection exists in the two parameters. As for ***Spk*** and ***Vmp***, both of them represent peak features above the core surface so that it is reasonable to put one of them into **CPS** [13]. ***Vvv*** and ***Svk*** characterize surface valley. ***Spd*** and ***Spc*** comprehensively describe the average surface peak density and peak curvature radius. They are the characteristic descriptions of different asperity peaks on the surface. ***Sxp*** and ***Smr1*** can be used to help define other roughness parameters, such as ***Sk***, ***Svk***, etc. [35].

### 3.2. Establishment of Optimal Explicit Expression

Although the separation of **CPS** and **RPS** is achieved through the classification method, it is still unknown how **CPS** represents the information in **RPS**. Besides, it has not been verified whether **CPS** can predict all parameters and thus achieve comprehensive characterization of surface topography. Therefore, only through the mapping relationship between **CPS** and **RPS** and the establishment of a quantitative model between them, **CPS** has the capability of topography characterization under the control of all roughness parameters.

#### 3.2.1. Core Roughness Parameters in CPS

In order to meet the needs of industrial production, the paper sets out from the polynomial regression model with strong nonlinear ability and designs a new method of items reduction to establish the optimal explicit expression between **CPS** and **RPS**. After selection, there are 12 parameters in **CPS** and 14 parameters in **RPS**. If the parameters in **CPS** are regarded as independent variables and each parameter in **RPS** is treated as the dependent variable, the number of polynomial terms will explode, and it is easy to overfit. Even if the pruning method is introduced at this time, the efficiency of solving the optimal expression will be greatly reduced.

In addition, combined with the definitions, not every redundant parameter needs all parameters in **CPS** to characterize their information.

**Figure 6:**
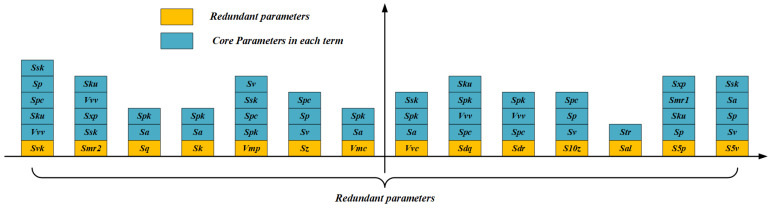
CRP.

According to the statistical regression theory and the interpretation of the independent variables by Friend et al. [36], the independent variables in the regression model should minimize the collinearity. Collinear variables do not increase the fit of the model. Then Yang et al. [22] put forward the principle of “non-strong and non-weak” to find the core independent variables with low collinearity, which have strong correlation with the dependent variable, but are relatively independent. In this study, the principle of “non-strong and non-weak” is introduced to screen the **CRP** and establish the optimal explicit expression. Figure 6 shows **CRP** corresponding to **RPS**. The number and types of **CRP** are extremely different, which is consistent with the actual cognition.

#### 3.2.2. Optimal Explicit Expression

Although the core roughness parameters to characterize **RPS** are further determined with the principle of “non-strong and non-weak”, it can be seen from Figure 5 that the definition and formula of ***Sp*** is Sp+Sv=Sz, but the **CRP** of ***Sz*** are ***Sp***, ***Sv*** and ***Spc***. The **CRP** of ***Sq*** are ***Sa*** and ***Spk***. It is different from other works. The core parameter of ***Sal*** is ***Str***, and they are consistent with the definitions. This reveals that the judgement method only by the principle of “non-strong and non-weak” will lead to the situation that some parameters still have information confusion and that the results are not reliable enough. Therefore, the paper designs a new redundant item pruning method to automatically identify and find roughness parameters with strong characterization ability with reliability.

Since the roughness parameter sets with clear equality are ***Sz***, ***Sp*** and ***Sv*** (Sp+Sv=Sz), the research will discuss these three parameters in detail to verify the reliability and stability of the redundancy information reduction technique.

At the beginning, ***Sz*** is introduced to the dependent variable, while the core parameters of ***Sp***, ***Sv*** and ***Spc*** in Figure 6 are regarded as independent variables. With the coefficients in model (9) solved, the relative error MRE will be obtained. After that, the polynomial of ***Sz*** will be addressed by the designed redundant item pruning method. Table 4 records the deleted item and the relative error change in the whole process, where 0 means the item is eliminated and 1 means retained.

In the pruning process of ***Sz***, the first item to be eliminated is ***Spc***, then ***Spc***^2^, ***Sv*** * ***Spc*** and ***Sp*** * ***Spc***. All of them contain ***Spc***. Combined with the parameter definition, the pruning process shows that the method can preferentially identify and eliminate items with low correlation with ***Sz***. The method is highly reliable and can actively discriminate the roughness parameters with strong characterization ability. In addition, on account of the error jump in 09, the 08 is selected as the optimal explicit expression at the end: $Sz=Sp+Sv+6.2654\times 10^{-8}$, and the relative error is $2.0543\times 10^{-7}$.

Compared with the real formula Sp+Sv=Sz, the optimal explicit expression is consistent with the real one. The pruning method ensures the reliability of the accuracy. At the same time, the influence of ***Spc*** is removed through continuous deletion. It verifies the ability of the proposed pruning method to remove the chaotic representation of parameters and greatly reduce the number of terms. The method is suitable for the optimal explicit expression.

After the accuracy of ***Sz*** is verified, each parameter in **RPS** gets a similar treatment. The rank of the core parameters is shown in Table 5(a). Due to the large number and types in **CRP**, it is bound to cause confusion in the model. Therefore, the quadratic square terms are sorted in order $x_{1}^{2},x_{2}^{2},\ldots x_{n}^{2}$, and then the cross terms $x_{1}x_{2},x_{1}x_{3},\ldots ,x_{1}x_{n},x_{2}x_{3},\ldots ,x_{2}x_{n},\ldots x_{n-1}x_{n}$ are arranged as above. Finally, sort the first order items and add the constant item at the end. The optimal term for each redundant parameter expression is selected in Table 5(b).

As ***Sz*** has been analyzed in the previous section, only other parameters are further elaborated here. For ***Sq***, the largest coefficient term in the optimal expression is the first-order ***Sa***, and the coefficients about ***Sv*** and ***Spk*** are all relatively smaller. It shows that ***Sq*** is mainly regulated by the primary term ***Sa***, while the surface peak-valley extreme features controlled by ***Sv*** and ***Spk*** account for a relatively low proportion in the information characterization of ***Sq***.

This also explains why ***Sa*** and ***Sq*** are strongly correlated, but not completely correlated. ***Sal*** and ***Str*** present a completely linear expression, conforming to the definitions of these two parameters. In ***Sk***, it is expressed by the first term of ***Sa*** and ***Spk***, indicating that the surface core height is actually characterized by the surface arithmetic mean height and the protruding peak height, and the influence of ***Sa*** is greater.

For *Svk*, it still retains quite a number of terms. The largest coefficient is the first-order term ***Vvv***, illustrating that ***Vvv*** has a good ability to characterize the surface valley features defined by ***Svk***, but at the same time it needs other parameters to realize the additional information of ***Svk***. In the optimal explicit expression of ***Smr2***, the quadratic cross term and the first term have larger coefficients. It indicates that ***Smr2*** is mainly affected by the coupling effect of surface valley void volume, surface skewness, kurtosis, and peak maximum height. These four reflect the surface bearing ability from different angles.

However, ***Vvc*** and ***Vmc*** are mainly controlled by ***Sa***, verifying the highest frequency of ***Sa*** in industrial applications. ***Vmp*** is mainly affected by the independent characterization of ***Spk*** and the coupled characterization of surface protruding peak height and skewness. The independent characterization of the first order ***Spk*** is stronger than the coupled characterization of the surface protruding peak height and skewness. It explains why ***Vmp*** and ***Spk*** are strongly correlated but with a little redundant information.

**Figure 7:**
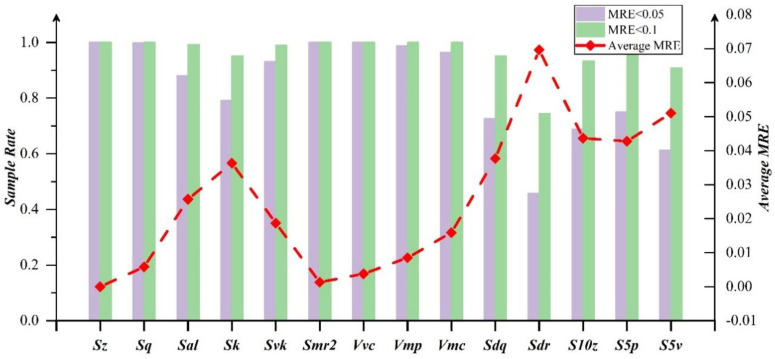
Parameter error distribution of optimal explicit expression.

For the optimal explicit expression of ***Sdq***, the final retained coefficients are relatively evenly distributed, indicating that ***Sdq*** covers a wide range of information and is defined by different features of the surface. In ***Sdr***, all items of ***Spk*** are removed, indicating that the height of the surface protruding peak has little effect on it. The surface extremum features covered by ***S10z***, ***S5p*** and ***S5v*** can be characterized with the coupling of ***Sp***, ***Sv*** and other parameters. In order to better distinguish the characterization ability of the optimal explicit expression, Figure 7 reveals the sample error distribution and average relative error of each redundant parameter in 1000 reconstructed surfaces. In the 1000 surfaces, the relative error of **RPS** is less than 0.1 and the proportion of samples is more than 90% except for ***Sdr***. Some parameters even reach 100%. For the surface proportion with a relative error less than 0.05, the remaining are basically above 80%, but ***Sdq***, ***Sdr***, ***S10z***, ***S5p*** and ***S5v*** drop to a large extent. In addition, the maximum average relative error of the parameters is 0.07 and the rest of the parameters are basically within 0.05 from the broken line distribution. It indicates that **CPS** can achieve full coverage of the surface topography information and remove the redundancy through the optimal expression.

### 3.3. Experiment Verification

Although **RPS** and **CPS** have been distinguished with the help of 1000 reconstructed surfaces and the optimal explicit expression of **RPS** has been established, the reconstructed surfaces are generated by the mathematical model and since factors such as tool runout and measurement errors cannot be avoided in the real machining process, the real surface has more randomness in the height distribution. Therefore, after the preliminary theoretical exploration is carried out with the reconstructed surfaces, it is necessary to verify the reliability of the method and analysis with the real grinding surfaces. The experiment conditions are shown in Table 6.

**Figure 8:**
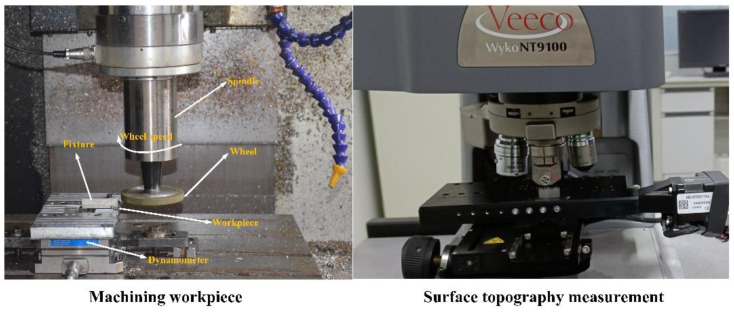
Machining and topography measurement.

The component is 12Cr2Ni4A steel, the surface roughness morphology gets measured with the white light interferometer Wyko NT9100, the sampling area is 0.48 mm × 0.64 mm = 0.3072 mm^2^, and the sampling interval is 1 µm. The machining process and 3D roughness topography measurement are shown in Figure 8.

A total of 44 surfaces were collected through the grinding experiment, and 26 surface roughness parameters were substituted into the optimal explicit expression of **RPS** to verify its generalization and reliability.

**Figure 9:**
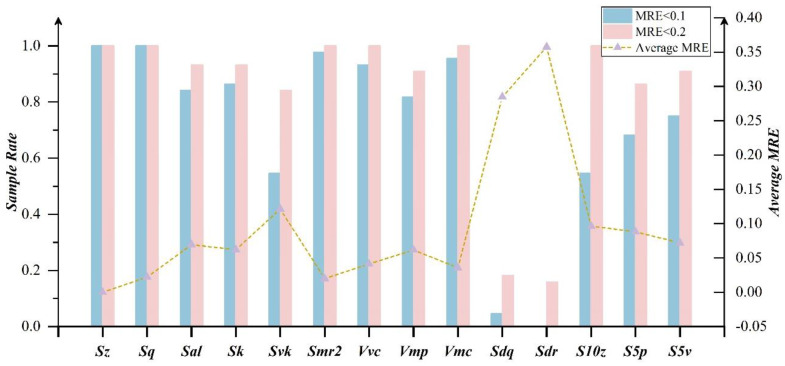
Parameter error distribution of experiment surfaces.

It can be seen from Figure 9 that parameters ***Svk***, ***S10z***, ***S5p*** and ***S5v*** with the relative errors less than 0.1 mainly account for 60–70%. However, with the relative errors less than 0.2, the proportion of these parameters has increased significantly, basically reaching about 0.9. Besides, the average error of parameters other than ***Sdq*** and ***Sdr*** is basically within 10%, indicating that the optimal explicit expressions of the remaining parameters are still reliable.

However, as ***Sdq*** and ***Sdr*** present such a large deviation, further research must be considered. The two are significantly different from others in terms of the relative error proportional distribution and average relative error. ***Sdr*** of the reconstructed surface in Figure 7 also belongs to the maximum error term. Considering that there are some slight differences between the reconstructed surface and the measured surface, the mathematical model cannot realize the topography feature control defined by all roughness parameters. The accumulation of these factors further exacerbates the degradation of the optimal expression prediction ability about ***Sdq*** and ***Sdr***.

In addition, the correlation coefficient of ***Sdq*** and ***Sdr*** is as high as 0.9843, and the two show a strong linear correlation. One should be selected into **CPS** and the other gets into **RPS**. Taking ***Sa*** as the benchmark, the correlation coefficients between ***Sdq*** and ***Sdr*** to ***Sa*** are 0.829 and 0.806, respectively. ***Sdr*** with small correlation with ***Sa*** (less repetition of topographic information with ***Sa***) is selected into **CPS**, and ***Sdq*** belongs to **RPS**.

Then the “non-strong and non-weak” principle and “polynomial pruning method” are used to find the core parameters and the optimal explicit expression is constructed. The core parameters of ***Sdq*** are ***Sdr***, ***Sxp***, ***Sv*** and ***Sp***, and the optimal expression constructed is as follows:

***Sdq*** = −4.5972 × 10^−5^***Sp***^2^ − 1.5786 × 10^−5^***Sv***^2^ − 0.0141***Sxp***^2^ − 0.0012***Sdr***^2^ + 9.1066 × 10^−5^***SpSv*** + 0.002***SpSxp*** − 0.0027***SvSxp*** + 1.9366 × 10^−4^***SvSdr*** − 0.0019***SxpSdr*** − 0.0035***Sp*** + 0.005***Sv*** + 0.0673***Sxp*** + 0.0517***Sdr*** + 0.0346

The adjusted optimal expression of ***Sdq*** can achieve the accuracy of approximately 0.9 on the measured surface with high reliability. So far, combined with theoretical and experiment analysis, the number of parameters in **CPS** to truly characterize and control surface roughness morphology is 13. The method achieves 50% reduction in the overall roughness parameters and builds a rational characterization model with fewer parameters to describe the integrity of the surface features.

### 3.4. Significance of CPS for Surface Performance

The establishment of **RPS** and **CPS** and the optimal explicit expressions clarify the correlation between roughness parameters and realize the complete characterization of surface features with fewer parameters. The method finds the parameters truly controlling the surface roughness and provides guidance for researchers to apply, but these analyses are still limited to the internal characterization of surface geometry and do not discuss about the relationships between **CPS** and surface performance. The roughness parameter that can well characterize surface performance is the focus of industry and research.

Different performance parameters are always closely related to the surface geometry topography characteristics described by roughness parameters. Owing to the large number of performance parameters and the complex correlation between the 26 roughness parameters, the existing performance screening and characterization parameter method [22] will result in the explosion of the final characterization parameters and unreliability. For example, the selected parameter is actually regulated by other roughness parameters, which will lead to deviations from the expected target, so that the better characterization effect is more likely to lose. **CPS** and **RPS** proposed in this paper can solve the above issues.

**Figure 10:**
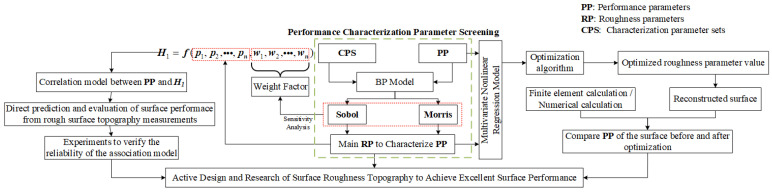
The method of correlation between **CPS** and surface performance.

Since **CPS** can realize the complete description of the surface topography information, when the correlation between surface performance and all roughness parameters is studied, more attention should be paid to observe the correlation between the surface performance and **CPS**. The topographic features, mainly influencing the surface performance, can be identified by selecting the parameters from **CPS**. Therefore, a method is designed to study the correlation between **CPS** and surface performance and to explain the significance of **CPS** in Figure 10.

The research focuses on the geometric characterization of the rough surface and clarifies the correlation among roughness parameters, so the correlation between **CPS** and surface performance belongs to the further expansion of the research. The method of correlation between **CPS** and surface performance will help prove the basic significance and applicability of this research. It will enable researchers to better understand the engineering significance of **CPS** for the realization in co-design and manufacture of rough surfaces. Therefore, here only provides a feasibility assessment and a rough introduction to the correlation method between **CPS** and surface performance.

(1) Regarding the performance characterization screening method described in the green box at the core position in Figure 10, this part belongs to the improvement and expansion of the method proposed in the literature [22] to screen the main roughness parameters based on the contact performance. Literature [22] mentions that since there is no direct physical model for roughness parameters and contact stress, the BP neural network model is introduced to establish the mapping relationship between the two, and then the main roughness parameters affecting the contact stress are identified by the quantitative **Sobol** and qualitative **Morris** analysis in the sensitivity analysis. The BP neural network has universality in fitting continuous data and different performance parameters belong to continuous data, so the extension of its contact performance to different performance parameters in this section will not affect the reliability of the method. In addition, the method replaces 26 roughness parameters with **CPS**, which will make the final parameters used to characterize the surface performance more accurate. Then by substituting different performance parameters and counting the frequency of different selected parameters in **CPS**, the ability of roughness parameters to characterize various performance characteristics will be distinguished;
(2) On the right side of the green box in Figure 10, the work of establishing the multivariate nonlinear regression model between the performance parameters and the main roughness parameters in **CPS** is easy to be completed with the help of the polynomial nonlinear regression and pruning techniques proposed in the paper. Regarding the inverse optimization of the explicit regression model, it is not difficult to find the suitable optimization algorithm to get the best parameter range. Although it is difficult to control the roughness parameters in the actual surface machining, the surface reconstruction algorithm will be an ideal way to realize it.

For the generation of reconstructed surfaces with specified roughness parameters, the literature [10] proposed a reliable method. The contact fatigue calculation model of rough surfaces, such as KE finite element model and Wen’s numerical calculation model [37], can complete the performance prediction in the reconstructed surface.

This part belongs to theoretical research based on mathematical models and contact theory. It has the advantages of low experimental cost and high efficiency and can provide paths and basic guidance for finding and designing the suitable rough surface with excellent performance;

(3) On the left side of the green box in Figure 10, it focuses on experiment research and verification. Even if the same batch of workpieces (surface residual stress, hardness and other material properties are considered to be nearly the same) are under the same loading conditions, the influence of other surface features in addition to the morphology features defined by ***Sa*** will still lead to great differences in contact properties, friction and wear, fatigue and other properties. However, the producers cannot judge the quality of the same batch, which will greatly reduce the service performance and increase the production cost of the enterprise.

This section will solve the above problems. At first, the weight factors of **CPS** are extracted, and the function $H_{1}=f(p_{1},p_{2},\ldots ,p_{n},w_{1},w_{2},\ldots ,w_{n})$ is constructed by combining the selected roughness parameters in **CPS**. The purpose of this step is to facilitate the observation and analysis about the comprehensive influence of the selected parameters on performance parameters. If only based on a single parameter, it will inevitably lead to the incompleteness of information. Secondly, the way to establish a correlation model between performance parameters and ***H*_1_** is more convenient for experiment verification.

Due to the huge experiment cost, if the multiple regression analysis on the right side is carried out, a large amount of experiment is required to get an accurate and reliable model. However, the purpose of the experiment research in this part is to rank the surface quality of different workpieces from the selected roughness parameters in **CPS**, so a particularly accurate model is not a must. Therefore, the feasibility of this part based on experiments is extremely high.

(4) Whether it is to screen the performance characterization parameters, or to establish an accurate nonlinear regression model from the theoretical view, or to evaluate the characterization parameters through experimental research, the establishment of the initial **CPS** is indispensable. Due to the continuous accumulation of errors, if the correct **CPS** cannot be obtained or they are selected only by experience, the correlation analysis between **CPS** and the surface performance will inevitably be unreliable.

## 4. Conclusions

(1) Based on 1000 reconstructed surfaces, 26 roughness parameters are roughly classified into **CPS** and **RPS** by Pearson correlation analysis. The principle of “non-strong and non-weak” helps **CPS** extract key factors from **CRP** to facilitate the establishment of subsequent expressions. The results demonstrate that the **RPS** information can be covered by **CPS**;
(2) The optimal explicit expressions of **CPS** and **RPS** get established, and the accuracy is basically above 90%. Then a polynomial pruning method is designed to find roughness parameters with strong ability to characterize surface information. The correlation between **CPS** and **RPS** is quantified to clarify the cause of application confusion. The results show **RPS** is independently affected and coupled by several different core parameters;
(3) The experiment verifies the reliability of the optimal explicit expression of **RPS** and surface characterization method and helps fix the number in **CPS** at 13. They are ***Sa***, ***Ssk***, ***Sku***, ***Sp***, ***Sv***, ***Str***, ***Spk***, ***Smr1***, ***Sxp***, ***Vvv***, ***Spd***, ***Spc*** and ***Sdq***. **RPS** accounts for 50% of the overall roughness parameter set, and the method realizes the comprehensive description of surface features with a smaller number of parameters, which has been well verified by the theoretical and experimental analysis;
(4) A surface characterization method for screening **CPS** is designed to find the key factors that really control the surface morphology. It also solves the dilemma of blindly or empirically selecting roughness parameters in industrial production. The reliability of the method to explore the correlation between **CPS** and different surface performance parameters is analyzed in detail. It proves the engineering significance of **CPS** for realizing co-design and manufacture in rough surfaces.

## Figures and Tables

**Table 1:** Roughness Parameters.

Category	Symbol	Definition
Height Parameters	***Sa***	Arithmetical mean height
	***Sz***	Maximum height
	***Sq***	Root mean square height
	***Ssk***	Skewness
	***Sku***	Kurtosis
	***Sp***	Maximum peak height
	***Sv***	Maximum pit depth
Hybrid parameters	***Sdq***	Root mean square gradient
	***Sdr***	Developed interfacial area ratio
Feature parameters	***Spd***	Density of peaks
	***Spc***	Arithmetic mean peak curvature
	***S5p***	Five-point peak height
	***S5v***	Five-point pit height
	***S10z***	Ten-point height of surface
Functions parameters	***Sk***	Core height
	***Spk***	Reduced peak height
	***Svk***	Reduced valley height
	***Smr1***	Material ratio in peak
	***Smr2***	Material ratio in valley
	***Sxp***	Peak extreme height
Volume parameters	***Vmp***	Peak material volume
	***Vmc***	Core material volume
	***Vvc***	Core void volume
	***Vvv***	Dale void volume
Space parameters	***Sal***	Autocorrelation length
	***Str***	Texture aspect ratio

**Table 2:** Reconstructed coefficients.

Reconstructed Coefficients							
	*k*_1_	*k*_2_	*k*_3_	*a*_1_	*a*_2_	*a*_3_	*a*_4_
Minimum	0.4	−5.5	0.6	0.6	0.08	0.08	0.0005
Maximum	3.4	2.5	50	0.9	0.12	0.12	0.0012

**Table 3:** Correlation judgment criteria.

Range	Conclusion
$0\leq \left|r_{ij}\right|<0.1$	Very weakly correlated
$0\leq \left|r_{ij}\right|<0.3$	Weakly correlated
$0.3\leq \left|r_{ij}\right|<0.7$	Moderately correlated
$0.7\leq \left|r_{ij}\right|<0.9$	Strongly correlated
$0.9\leq \left|r_{ij}\right|<1$	Very strongly correlated

**Table 4:** Polynomial pruning of ***Sz***.

Dependent Variable	Num	Polynomial terms										MRE
		*Sp^2^*	*Sv^2^*	*Spc^2^*	Sp * Sv	Sp * Spc	Sv * Spc	*Sp*	*Sv*	*Spc*	Const	
***Sz***	01	1	1	1	1	1	1	1	1	1	1	2.1205 × 10^−7^
	02	1	1	1	1	1	1	1	1	0	1	2.1259 × 10^−7^
	03	1	1	0	1	1	1	1	1	0	1	2.1243 × 10^−7^
	04	1	1	0	1	1	0	1	1	0	1	2.1147 × 10^−7^
	05	1	1	0	1	0	0	1	1	0	1	2.1036 × 10^−7^
	06	0	1	0	1	0	0	1	1	0	1	2.0950 × 10^−7^
	07	0	0	0	1	0	0	1	1	0	1	2.0807 × 10^−7^
	08	0	0	0	0	0	0	1	1	0	1	2.0543 × 10^−7^
	09	0	0	0	0	0	0	1	1	0	0	1.4 × 10^−3^

**Table 5:** Part (a) Rank of core parameters in **RPS**; Part (b) Optimal explicit expression of **RPS**.

Core Parameters in RPS													
(a)													
*Sz*	*Sq*	*Sal*	*Sk*	*Svk*	*Smr2*	*Vvc*	*Vmp*	*Vmc*	*Sdq*	*Sdr*	*S10z*	*S5p*	*S5v*
***x***_1_ = ***Sp***	***x***_1_ = ***Sa***	***x***_1_ = ***Str***	***x***_1_ = ***Sa***	***x***_1_ = ***Ssk***	***x***_1_ = ***Ssk***	***x***_1_ = ***Sa***	***x***_1_ = ***Ssk***	***x***_1_ = ***Sa***	***x***_1_ = ***Sku***	***x***_1_ = ***Spk***	***x***_1_ = ***Sp***	***x***_1_ = ***Sku***	***x***_1_ = ***Sa***
***x***_2_ = ***Sv***	***x***_2_ = ***Sv***		***x***_2_ = ***Spk***	***x***_2_ = ***Sku***	***x***_2_ = ***Sku***	***x***_2_ = ***Ssk***	***x***_2_ = ***Sv***	***x***_2_ = ***Spk***	***x***_2_ = ***Spk***	***x***_2_ = ***Vvv***	***x***_2_ = ***Sv***	***x***_2_ = ***Sp***	***x***_2_ = ***Ssk***
***x***_3_ = ***Spc***	***x***_3_ = ***Spk***			***x***_3_ = ***Sp***	***x***_3_ = ***Sxp***	***x***_3_ = ***Spk***	***x***_3_ = ***Spk***		***x***_3_ = ***Vvv***	***x***_3_ = ***Spc***	***x***_3_ = ***Spc***	***x***_3_ = ***Smr1***	***x***_3_ = ***Sp***
				***x***_4_ = ***Vvv***	***x***_4_ = ***Vvv***		***x***_4_ = ***Spc***		***x***_4_ = ***Spc***			***x***_4_ = ***Sxp***	***x***_4_ = ***Sv***
				***x***_5_ = ***Spc***									
**(b)**													
***Sz***	***Sq***	***Sal***	***Sk***	***Svk***	***Smr2***	***Vvc***	***Vmp***	***Vmc***	***Sdq***	***Sdr***	***S10z***	***S5p***	***S5v***
0	0	0	0	−3.86 × 10^−2^	6.58 × 10^−1^	−1.01 × 10^−1^	−2.15 × 10^−3^	0	−1.97 × 10^−4^	0	−1.49 × 10^−2^	−1.47 × 10^−2^	0
0	−1.66 × 10^−4^	4.08 × 10^2^	0	−1.17 × 10^−3^	2.07 × 10^−2^	5.00 × 10^−2^	−1.20 × 10^−5^	0	0	0	−1.07 × 10^−2^	−1.29 × 10^−2^	0
0	1.97 × 10^−1^	2.05	0	−1.40 × 10^−4^	−1.13 × 10^−1^	−2.07 × 10^−1^	0	0	0	1.61 × 10^−2^	−1.44 × 10^−2^	−1.53 × 10^−2^	−2.64 × 10^−3^
0	6.64 × 10^−3^		3.43	0	0	3.47 × 10^−1^	1.97 × 10^−5^	1.22	−2.54 × 10^−4^	0	2.30 × 10^−3^	−4.31 × 10^−1^	−1.55 × 10^−2^
0	−1.90 × 10^−1^		−4.67 × 10^−1^	4.03 × 10^−5^	1.06 × 10^−1^	3.16 × 10^−1^	−2.24 × 10^−4^	−1.69 × 10^−1^	2.64 × 10^−3^	0	1.64 × 10^−2^	−8.62 × 10^−4^	0
0	−1.31 × 10^−3^		−1.15 × 10^−2^	2.38 × 10^−3^	−2.10	−2.24 × 10^−1^	1.57 × 10^−2^	−1.81 × 10^−3^	4.94 × 10^−2^	1.62	1.57 × 10^−2^	7.35 × 10^−2^	0
1	1.20			2.26 × 10^−3^	−1.78 × 10^1^	1.62	−5.85 × 10^−4^		−4.70 × 10^−4^	0	7.13 × 10^−1^	1.05 × 10^−1^	2.08 × 10^−1^
1	5.78 × 10^−3^			0	2.41 × 10^−1^	4.43 × 10^−2^	1.46 × 10^−3^		0	−3.04	7.30 × 10^−1^	−2.21 × 10^−2^	−5.04 × 10^−2^
0	5.35 × 10^−2^			−4.78 × 10^−3^	−2.40	−1.85 × 10^−1^	−4.40 × 10^−5^		0	2.96 × 10^−2^	2.14 × 10^−1^	1.55 × 10^−1^	−1.35 × 10^−1^
6.27 × 10^−8^	−4.31 × 10^−5^			−2.74 × 10^−4^	0	1.29 × 10^−3^	−8.31 × 10^−4^		3.68 × 10^−2^	1.54 × 10^−2^	2.09 × 10^−2^	1.54 × 10^−1^	3.84 × 10^−3^
				0	2.69		−1.47 × 10^−3^		4.30 × 10^−3^			−4.10 × 10^−1^	−1.07 × 10^−1^
				5.41 × 10^−4^	−6.05 × 10^−1^		−1.12 × 10^−4^		−6.40 × 10^−3^			5.39 × 10^−1^	1.98 × 10^−1^
				0	4.87		9.30 × 10^−2^		−1.88 × 10^−1^			−6.40 × 10^−2^	1.54 × 10^−1^
				1.48 × 10^−4^	−8.12 × 10^1^		5.21 × 10^−4^		2.61 × 10^−2^			1.12 × 10^−2^	6.11 × 10^−1^
				0	9.14 × 10^1^		5.93 × 10^−5^		−4.46 × 10^−3^			9.52 × 10^−1^	2.34 × 10^−1^
				−4.83 × 10^−2^									
				2.82 × 10^−2^									
				6.56 × 10^−3^									
				4.71									
				−7.66 × 10^−3^									
				−1.01 × 10^−1^									

**Table 6:** Experiment conditions.

Machining Parameters	Parameter Values
Grinding wheel	CBN grinding wheel
Wheel radius	100 mm
Wheel mesh	120
Wheel speed	500–3000 r/min
Cutting speed	200 mm/min
Cutting depth	5–30 µm

## Data Availability

All data that support the findings of this study are included within the article.
